# Crystal structure of (*E*)-2-benzyl­idene-4-[(3-phenyl-4,5-di­hydro­isoxazol-5-yl)meth­yl]-2*H*-benzo[*b*][1,4]thia­zin-3(4*H*)-one

**DOI:** 10.1107/S2056989015009755

**Published:** 2015-05-23

**Authors:** Nada Kheira Sebbar, Mohamed Ellouz, El Mokhtar Essassi, Mohamed Saadi, Lahcen El Ammari

**Affiliations:** a Laboratoire de Chimie Organique Hétérocyclique URAC 21, Pôle de Compétence Pharmacochimie, Av. Ibn Battouta, BP 1014, Faculté des Sciences, Université Mohammed V, Rabat, Morocco; b Moroccan Foundation for Advanced Science, Innovation and Research (MASCIR), Rabat Design Center, Rue Mohamed Al Jazouli, Madinat El Irfane, 10100 Rabat, Morocco; c Laboratoire de Chimie du Solide Appliquée, Faculté des Sciences, Université Mohammed V, Avenue Ibn Battouta, BP 1014, Rabat, Morocco

**Keywords:** crystal structure, benzo­thia­zine, di­hydro­isoxazole, C—H⋯O,N hydrogen bonding

## Abstract

In the title compound, C_25_H_20_N_2_O_2_S, the di­hydro­isoxazole ring exhibits an envelope conformation with the methine atom being the flap, while the 1,4-thia­zine ring displays a screw-boat conformation. The six-membered ring fused to the 1,4-thia­zine ring makes dihedral angles of 63.04 (2) and 54.7 (2)° with the mean planes through the five-membered heterocycle and the attached phenyl ring, respectively. The phenyl group connected to the 1,4-thia­zine ring is disordered over two sites [major component = 0.57 (2)]. The most prominent inter­actions in the crystal structure are C—H⋯O hydrogen bonds that link mol­ecules, forming inversion dimers, and C—H⋯N hydrogen bonds that link the dimers into columns parallel to the *b* axis.

## Related literature   

For the biological activity and pharmaceutical properties of benzo­thia­zines and their derivatives, see: Fringuelli *et al.* (1998 ▸); Rathore & Kumar (2006 ▸); Baraza­rte *et al.* (2008 ▸); Bakavoli *et al.* (2007 ▸). For related structures, see: Saeed *et al.* (2010 ▸); Afrakssou *et al.* (2011 ▸); Sebbar *et al.* (2014*a* ▸,*b* ▸).
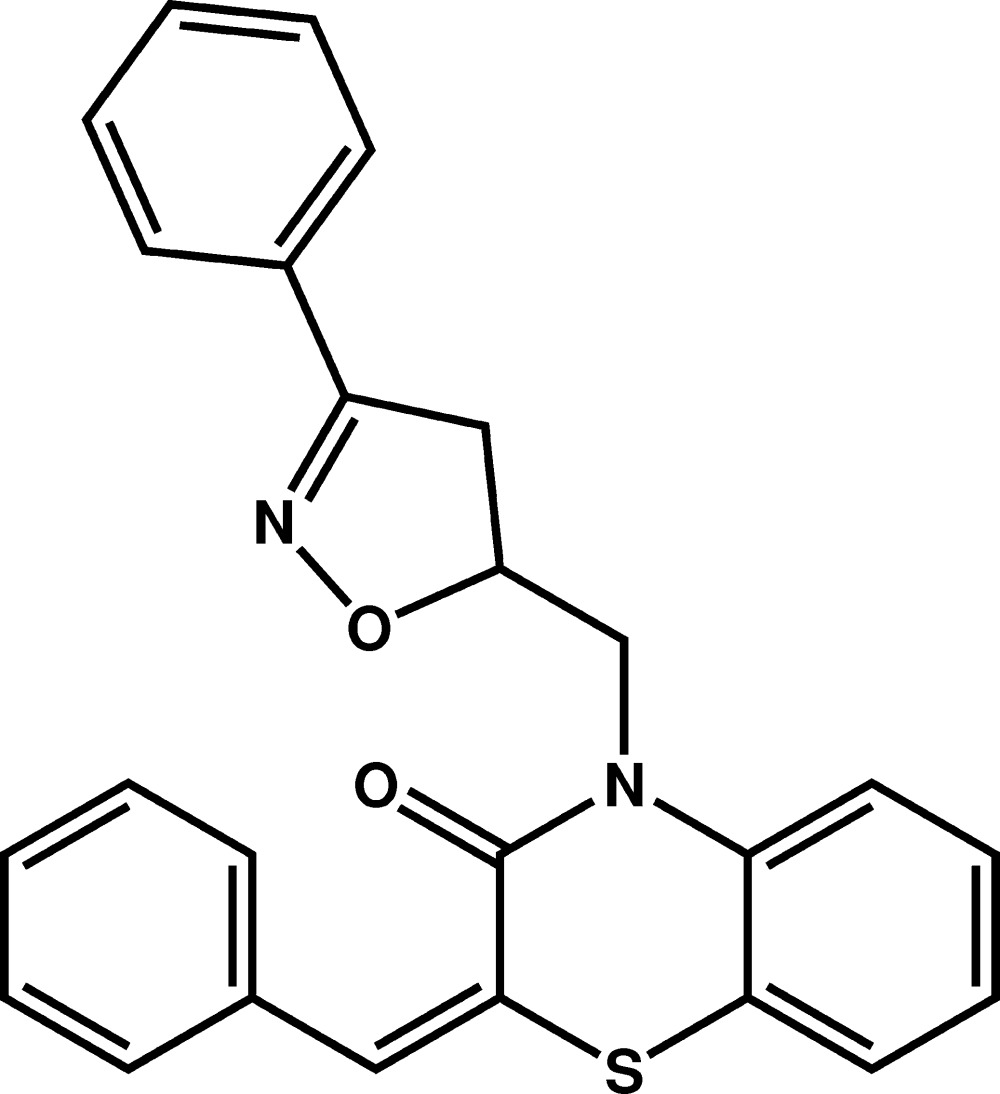


## Experimental   

### Crystal data  

C_25_H_20_N_2_O_2_S
*M* *_r_* = 412.49
Monoclinic, 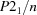

*a* = 17.4463 (16) Å
*b* = 5.3024 (4) Å
*c* = 22.778 (2) Å
β = 106.370 (5)°
*V* = 2021.7 (3) Å^3^
*Z* = 4
Mo *K*α radiation
μ = 0.19 mm^−1^
*T* = 296 K
0.36 × 0.31 × 0.26 mm

### Data collection  

Bruker X8 APEX diffractometer
Absorption correction: multi-scan (*SADABS*; Bruker, 2009 ▸) *T*_min_ = 0.504, *T*_max_ = 0.748
27864 measured reflections
4149 independent reflections
1980 reflections with *I* > 2σ(*I*)
*R*_int_ = 0.095

### Refinement  

*R*[*F* ^2^ > 2σ(*F* ^2^)] = 0.057
*wR*(*F* ^2^) = 0.145
*S* = 1.00
4149 reflections
321 parameters
H-atom parameters constrained
Δρ_max_ = 0.48 e Å^−3^
Δρ_min_ = −0.26 e Å^−3^

### 

Data collection: *APEX2* (Bruker, 2009 ▸); cell refinement: *SAINT* (Bruker, 2009 ▸); data reduction: *SAINT*; program(s) used to solve structure: *SHELXS97* (Sheldrick, 2008 ▸); program(s) used to refine structure: *SHELXL97* (Sheldrick, 2008 ▸); molecular graphics: *ORTEPIII* (Burnett & Johnson, 1996 ▸) and *ORTEP-3 for Windows* (Farrugia, 2012 ▸); software used to prepare material for publication: *PLATON* (Spek, 2009 ▸) and *publCIF* (Westrip, 2010 ▸).

## Supplementary Material

Crystal structure: contains datablock(s) I. DOI: 10.1107/S2056989015009755/tk5368sup1.cif

Structure factors: contains datablock(s) I. DOI: 10.1107/S2056989015009755/tk5368Isup2.hkl

Supporting information file. DOI: 10.1107/S2056989015009755/tk5368Isup3.cml

**Fig. 1.**
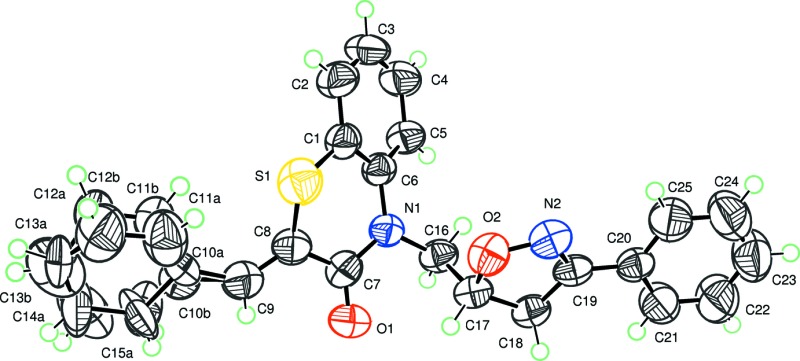
Molecular structure of the title compound with the atom-labelling scheme. Displacement ellipsoids are drawn at the 50% probability level. H atoms are represented as small circles. One phenyl ring is disordered over two positions.

. DOI: 10.1107/S2056989015009755/tk5368fig1.tif
Mol­ecular structure of the title compound with the atom-labelling scheme. Displacement ellipsoids are drawn at the 50% probability level. H atoms are represented as small circles. One phenyl ring is disordered over two positions.

CCDC reference: 1402017

Additional supporting information:  crystallographic information; 3D view; checkCIF report

## Figures and Tables

**Table 1:** Hydrogen-bond geometry (, )

*D*H*A*	*D*H	H*A*	*D* *A*	*D*H*A*
C21H21O1^i^	0.93	2.43	3.339(4)	166
C18H18*B*N2^ii^	0.97	2.56	3.526(3)	178

Symmetry codes: (i) 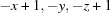; (ii) 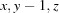.

## supplementary crystallographic information

### S1. Comment

Recently, a number of pharmacological tests revealed that 2*H*-1,4- benzothiazine derivatives present various biological activities including antifungal (Fringuelli *et al.*, 1998), antimicrobial (Rathore *et al.*, 2006), antimalarial (Barazarte *et al.*, 2008) and 15-lipoxygenase inhibition properties (Bakavoli *et al.*, 2007). In this work, we aim to prepare new derivatives of 3,4-dihydro-2*H*- benzo[*b*]1,4-thiazine for biological evaluation, as in the previous studies (Saeed *et al.*, 2010; Afrakssou *et al.*, 2011; Sebbar *et al.*, 2014*a*, 2014*b*. In the reaction, the oxime reacts with (*E*)-4-allyl-2-benzylidene-2*H*-benzo[*b*][1,4]thiazin-3(4*H*)-one in a biphasic medium (water-chloroform) at 0°C over 4 h to give a unique cycloadduct: (*E*)-2-benzylidene-4-((3-phenyl-4, 5-dihydroisoxazol-5-yl)methyl)-2*H*- benzo[*b*][1,4]thiazin-3(4*H*)-one (Scheme 1).

The molecule of the title compound is build up from two fused six-membered rings linked, *via* two –CH_2_– groups, on the one hand to a phenyl ring and on the other hand to the 3-phenyl-4,5-dihydroisoxazole system as shown in Fig. 1. The (C1 to C6) benzene cycle form dihedral angles of 63.04 (2)\ and 54.7 (2)° with the mean planes through the five-membered heterocycle and the attached phenyl ring, respectively. In the crystal, the molecules are linked by hydrogen bond (Table 1) in the way to build a dimers as shown in Fig. 2.

### S2. Experimental

To a solution of (*E*)-4-allyl-2-benzylidene-3,4-dihydro-2*H*- benzo[*b*]1,4-thiazine (1 g, 3.4 mmol) and benzaldoxime (0.81 ml, 6.8 mmol) in chloroform (30 ml) was added dropwise a 24% sodium hypochlorite solution (10 ml) at 273 K. Stirring was continued for 4 h. The organic layer was dried over Na_2_SO_4_ and the solvent was evaporated under reduced pressure. The residue was then purified by column chromatography on silica gel using a mixture of hexane/ethyl acetate (*v*/*v* = 80/20) as eluent. Colourless crystals were isolated when the solvent was allowed to evaporate (yield: 74%).

### S3. Refinement

The H atoms were located in a difference map and treated as riding with C—H = 0.93–0.98 Å, and with *U*_iso_(H) = 1.2 *U*_eq_. The phenyl group connected to the 1,4-thiazine ring is disordered over two sites [major component = 0.57 (2)].

### Crystal data

**Table:**

C_25_H_20_N_2_O_2_S	*F*(000) = 864
*M**_r_* = 412.49	*D*_x_ = 1.355 Mg m^−^^3^
Monoclinic, *P*2_1_/*n*	Mo *K*α radiation, λ = 0.71073 Å
*a* = 17.4463 (16) Å	Cell parameters from 4149 reflections
*b* = 5.3024 (4) Å	θ = 1.7–26.4°
*c* = 22.778 (2) Å	µ = 0.19 mm^−^^1^
β = 106.370 (5)°	*T* = 296 K
*V* = 2021.7 (3) Å^3^	Block, colourless
*Z* = 4	0.36 × 0.31 × 0.26 mm

### Data collection

**Table:**

Bruker X8 APEX diffractometer	4149 independent reflections
Radiation source: fine-focus sealed tube	1980 reflections with *I* > 2σ(*I*)
Graphite monochromator	*R*_int_ = 0.095
φ and ω scans	θ_max_ = 26.4°, θ_min_ = 1.7°
Absorption correction: multi-scan (*SADABS*; Bruker, 2009)	*h* = −21→21
*T*_min_ = 0.504, *T*_max_ = 0.748	*k* = −6→6
27864 measured reflections	*l* = −28→25

### Refinement

**Table:**

Refinement on *F*^2^	0 restraints
Least-squares matrix: full	Hydrogen site location: inferred from neighbouring sites
*R*[*F*^2^ > 2σ(*F*^2^)] = 0.057	H-atom parameters constrained
*wR*(*F*^2^) = 0.145	*w* = 1/[σ^2^(*F*_o_^2^) + (0.0437*P*)^2^ + 0.9738*P*] where *P* = (*F*_o_^2^ + 2*F*_c_^2^)/3
*S* = 1.00	(Δ/σ)_max_ = 0.008
4149 reflections	Δρ_max_ = 0.48 e Å^−^^3^
321 parameters	Δρ_min_ = −0.26 e Å^−^^3^

### Special details

**Table:**

Geometry. All e.s.d.'s (except the e.s.d. in the dihedral angle between two l.s. planes) are estimated using the full covariance matrix. The cell e.s.d.'s are taken into account individually in the estimation of e.s.d.'s in distances, angles and torsion angles; correlations between e.s.d.'s in cell parameters are only used when they are defined by crystal symmetry. An approximate (isotropic) treatment of cell e.s.d.'s is used for estimating e.s.d.'s involving l.s. planes.

### Fractional atomic coordinates and isotropic or equivalent isotropic displacement parameters (Å^2^)

**Table:**

	*x*	*y*	*z*	*U*_iso_*/*U*_eq_	Occ. (<1)
C1	0.17593 (18)	0.5680 (6)	0.36708 (14)	0.0498 (8)	
C2	0.1174 (2)	0.6377 (7)	0.31368 (19)	0.0693 (11)	
H2	0.0834	0.7719	0.3146	0.083*	
C3	0.1096 (2)	0.5102 (9)	0.25999 (18)	0.0784 (12)	
H3	0.0717	0.5614	0.2244	0.094*	
C4	0.1577 (2)	0.3074 (8)	0.25874 (16)	0.0707 (11)	
H4	0.1512	0.2177	0.2226	0.085*	
C5	0.21579 (19)	0.2357 (6)	0.31078 (14)	0.0543 (9)	
H5	0.2479	0.0968	0.3096	0.065*	
C6	0.22670 (17)	0.3703 (5)	0.36516 (14)	0.0418 (7)	
C7	0.28231 (19)	0.3227 (6)	0.47719 (16)	0.0529 (8)	
C8	0.21687 (19)	0.4767 (6)	0.48721 (15)	0.0542 (9)	
C9	0.1957 (2)	0.4326 (7)	0.53875 (16)	0.0659 (10)	
H9	0.2239	0.3061	0.5641	0.079*	
C10A	0.1328 (11)	0.561 (4)	0.5602 (8)	0.051 (3)	0.57 (2)
C11A	0.1042 (10)	0.781 (3)	0.5504 (5)	0.084 (3)	0.57 (2)
H11A	0.1229	0.8915	0.5258	0.101*	0.57 (2)
C12A	0.0437 (9)	0.861 (3)	0.5768 (6)	0.099 (4)	0.57 (2)
H12A	0.0224	1.0218	0.5680	0.118*	0.57 (2)
C13A	0.0161 (10)	0.713 (5)	0.6139 (12)	0.082 (6)	0.57 (2)
H13A	−0.0329	0.7447	0.6210	0.098*	0.57 (2)
C14A	0.0602 (15)	0.521 (5)	0.6399 (13)	0.094 (6)	0.57 (2)
H14A	0.0499	0.4360	0.6726	0.113*	0.57 (2)
C15A	0.1232 (12)	0.447 (3)	0.6169 (9)	0.090 (5)	0.57 (2)
H15A	0.1593	0.3255	0.6374	0.108*	0.57 (2)
C10B	0.1358 (18)	0.506 (5)	0.5683 (14)	0.060 (8)*	0.43 (2)
C11B	0.0671 (10)	0.674 (4)	0.5314 (8)	0.075 (5)	0.43 (2)
H11B	0.0663	0.7241	0.4920	0.090*	0.43 (2)
C12B	0.0078 (11)	0.752 (4)	0.5551 (8)	0.077 (5)	0.43 (2)
H12B	−0.0347	0.8505	0.5328	0.092*	0.43 (2)
C13B	0.0151 (18)	0.671 (8)	0.6186 (18)	0.108 (14)	0.43 (2)
H13B	−0.0142	0.7420	0.6428	0.129*	0.43 (2)
C14B	0.068 (3)	0.483 (9)	0.6378 (19)	0.17 (2)	0.43 (2)
H14B	0.0702	0.4082	0.6751	0.206*	0.43 (2)
C15B	0.1193 (14)	0.393 (7)	0.6073 (16)	0.142 (14)	0.43 (2)
H15B	0.1421	0.2345	0.6179	0.171*	0.43 (2)
C16	0.36034 (17)	0.1736 (5)	0.41243 (13)	0.0474 (8)	
H16A	0.3559	0.1496	0.3694	0.057*	
H16B	0.3637	0.0085	0.4313	0.057*	
C17	0.43583 (17)	0.3205 (5)	0.44203 (14)	0.0460 (8)	
H17	0.4441	0.3324	0.4863	0.055*	
C18	0.50908 (17)	0.2106 (5)	0.42840 (14)	0.0468 (8)	
H18A	0.5548	0.2102	0.4645	0.056*	
H18B	0.4994	0.0405	0.4124	0.056*	
C19	0.52051 (17)	0.3901 (5)	0.38119 (13)	0.0419 (7)	
C20	0.57826 (18)	0.3586 (5)	0.34590 (14)	0.0464 (8)	
C21	0.6339 (2)	0.1683 (7)	0.35988 (16)	0.0677 (10)	
H21	0.6341	0.0567	0.3914	0.081*	
C22	0.6896 (2)	0.1410 (8)	0.3275 (2)	0.0860 (13)	
H22	0.7278	0.0140	0.3380	0.103*	
C23	0.6888 (3)	0.2994 (8)	0.2803 (2)	0.0869 (13)	
H23	0.7264	0.2807	0.2587	0.104*	
C24	0.6332 (3)	0.4842 (8)	0.26472 (19)	0.0900 (13)	
H24	0.6323	0.5905	0.2321	0.108*	
C25	0.5777 (2)	0.5156 (7)	0.29715 (18)	0.0725 (11)	
H25	0.5398	0.6431	0.2862	0.087*	
N1	0.28861 (14)	0.3038 (4)	0.41850 (11)	0.0447 (6)	
N2	0.47581 (16)	0.5856 (4)	0.37434 (12)	0.0526 (7)	
O1	0.33070 (14)	0.2170 (5)	0.51952 (10)	0.0720 (7)	
O2	0.42730 (13)	0.5703 (3)	0.41480 (10)	0.0562 (6)	
S1	0.18110 (6)	0.72501 (16)	0.43621 (5)	0.0680 (3)	

### Atomic displacement parameters (Å^2^)

**Table:**

	*U*^11^	*U*^22^	*U*^33^	*U*^12^	*U*^13^	*U*^23^
C1	0.0428 (19)	0.0534 (19)	0.053 (2)	−0.0024 (16)	0.0122 (17)	0.0073 (16)
C2	0.053 (2)	0.078 (2)	0.074 (3)	0.011 (2)	0.013 (2)	0.031 (2)
C3	0.058 (3)	0.117 (3)	0.049 (3)	0.000 (2)	−0.002 (2)	0.031 (2)
C4	0.060 (2)	0.098 (3)	0.047 (2)	−0.008 (2)	0.0034 (19)	0.003 (2)
C5	0.048 (2)	0.062 (2)	0.047 (2)	−0.0018 (16)	0.0042 (17)	−0.0010 (18)
C6	0.0375 (18)	0.0419 (16)	0.044 (2)	−0.0032 (14)	0.0086 (15)	0.0042 (15)
C7	0.043 (2)	0.059 (2)	0.055 (2)	−0.0010 (17)	0.0116 (18)	0.0007 (18)
C8	0.050 (2)	0.063 (2)	0.046 (2)	−0.0028 (17)	0.0077 (17)	−0.0047 (17)
C9	0.056 (2)	0.084 (3)	0.054 (2)	0.007 (2)	0.0101 (19)	−0.002 (2)
C10A	0.056 (7)	0.063 (10)	0.035 (7)	0.004 (6)	0.016 (5)	0.006 (6)
C11A	0.085 (9)	0.094 (8)	0.081 (7)	−0.015 (7)	0.036 (6)	−0.027 (6)
C12A	0.087 (9)	0.114 (8)	0.095 (8)	0.000 (7)	0.025 (7)	−0.043 (7)
C13A	0.060 (9)	0.122 (14)	0.081 (13)	0.024 (9)	0.049 (9)	0.004 (9)
C14A	0.091 (11)	0.102 (9)	0.120 (17)	0.033 (8)	0.080 (11)	0.033 (9)
C15A	0.142 (14)	0.096 (7)	0.059 (9)	0.057 (7)	0.072 (9)	0.032 (6)
C11B	0.055 (9)	0.105 (11)	0.067 (8)	0.009 (8)	0.021 (7)	0.001 (8)
C12B	0.061 (9)	0.105 (11)	0.070 (10)	0.024 (8)	0.027 (7)	0.010 (8)
C13B	0.12 (2)	0.123 (19)	0.08 (2)	−0.070 (18)	0.028 (15)	−0.035 (17)
C14B	0.19 (3)	0.26 (4)	0.08 (2)	0.07 (2)	0.07 (2)	0.03 (2)
C15B	0.058 (12)	0.28 (4)	0.096 (17)	0.007 (15)	0.035 (10)	−0.01 (2)
C16	0.047 (2)	0.0446 (17)	0.046 (2)	0.0058 (15)	0.0060 (16)	−0.0037 (15)
C17	0.0451 (19)	0.0443 (17)	0.0435 (19)	0.0034 (15)	0.0042 (15)	−0.0010 (15)
C18	0.0439 (19)	0.0441 (17)	0.048 (2)	0.0064 (15)	0.0052 (15)	−0.0003 (15)
C19	0.0416 (18)	0.0341 (15)	0.0426 (19)	−0.0021 (14)	−0.0003 (15)	−0.0037 (14)
C20	0.0431 (19)	0.0451 (17)	0.047 (2)	−0.0032 (15)	0.0053 (16)	−0.0015 (16)
C21	0.072 (3)	0.070 (2)	0.066 (3)	0.019 (2)	0.028 (2)	0.0122 (19)
C22	0.082 (3)	0.091 (3)	0.095 (3)	0.030 (2)	0.042 (3)	0.013 (3)
C23	0.084 (3)	0.097 (3)	0.095 (3)	0.007 (3)	0.050 (3)	0.004 (3)
C24	0.097 (3)	0.096 (3)	0.089 (3)	0.005 (3)	0.046 (3)	0.027 (3)
C25	0.069 (3)	0.067 (2)	0.084 (3)	0.010 (2)	0.024 (2)	0.019 (2)
N1	0.0421 (15)	0.0516 (15)	0.0388 (16)	0.0008 (12)	0.0085 (13)	−0.0021 (12)
N2	0.0543 (17)	0.0399 (14)	0.0595 (19)	0.0019 (13)	0.0095 (15)	−0.0023 (13)
O1	0.0622 (16)	0.1030 (19)	0.0471 (15)	0.0177 (14)	0.0093 (13)	0.0139 (14)
O2	0.0586 (14)	0.0385 (12)	0.0724 (16)	0.0094 (11)	0.0200 (13)	−0.0006 (11)
S1	0.0727 (7)	0.0573 (5)	0.0770 (7)	0.0094 (5)	0.0258 (5)	−0.0015 (5)

### Geometric parameters (Å, º)

**Table:**

C1—C6	1.381 (4)	C11B—C12B	1.360 (15)
C1—C2	1.400 (4)	C11B—H11B	0.9300
C1—S1	1.761 (3)	C12B—C13B	1.48 (5)
C2—C3	1.370 (5)	C12B—H12B	0.9300
C2—H2	0.9300	C13B—C14B	1.35 (7)
C3—C4	1.369 (5)	C13B—H13B	0.9300
C3—H3	0.9300	C14B—C15B	1.36 (5)
C4—C5	1.378 (4)	C14B—H14B	0.9300
C4—H4	0.9300	C15B—H15B	0.9300
C5—C6	1.395 (4)	C16—N1	1.469 (3)
C5—H5	0.9300	C16—C17	1.515 (4)
C6—N1	1.424 (3)	C16—H16A	0.9700
C7—O1	1.224 (4)	C16—H16B	0.9700
C7—N1	1.376 (4)	C17—O2	1.453 (3)
C7—C8	1.473 (4)	C17—C18	1.514 (4)
C8—C9	1.347 (4)	C17—H17	0.9800
C8—S1	1.750 (3)	C18—C19	1.491 (4)
C9—C10B	1.45 (3)	C18—H18A	0.9700
C9—C10A	1.49 (2)	C18—H18B	0.9700
C9—H9	0.9300	C19—N2	1.280 (3)
C10A—C11A	1.26 (2)	C19—C20	1.465 (4)
C10A—C15A	1.48 (2)	C20—C21	1.373 (4)
C11A—C12A	1.418 (14)	C20—C25	1.386 (4)
C11A—H11A	0.9300	C21—C22	1.384 (5)
C12A—C13A	1.34 (3)	C21—H21	0.9300
C12A—H12A	0.9300	C22—C23	1.361 (5)
C13A—C14A	1.31 (5)	C22—H22	0.9300
C13A—H13A	0.9300	C23—C24	1.354 (5)
C14A—C15A	1.40 (3)	C23—H23	0.9300
C14A—H14A	0.9300	C24—C25	1.383 (5)
C15A—H15A	0.9300	C24—H24	0.9300
C10B—C15B	1.17 (4)	C25—H25	0.9300
C10B—C11B	1.54 (3)	N2—O2	1.418 (3)
			
C6—C1—C2	119.4 (3)	C14B—C13B—C12B	113 (3)
C6—C1—S1	121.0 (2)	C14B—C13B—H13B	123.4
C2—C1—S1	119.5 (3)	C12B—C13B—H13B	123.4
C3—C2—C1	120.6 (4)	C13B—C14B—C15B	126 (4)
C3—C2—H2	119.7	C13B—C14B—H14B	117.1
C1—C2—H2	119.7	C15B—C14B—H14B	117.1
C4—C3—C2	119.9 (3)	C10B—C15B—C14B	123 (4)
C4—C3—H3	120.0	C10B—C15B—H15B	118.6
C2—C3—H3	120.0	C14B—C15B—H15B	118.6
C3—C4—C5	120.3 (4)	N1—C16—C17	111.9 (2)
C3—C4—H4	119.8	N1—C16—H16A	109.2
C5—C4—H4	119.8	C17—C16—H16A	109.2
C4—C5—C6	120.4 (3)	N1—C16—H16B	109.2
C4—C5—H5	119.8	C17—C16—H16B	109.2
C6—C5—H5	119.8	H16A—C16—H16B	107.9
C1—C6—C5	119.2 (3)	O2—C17—C18	104.7 (2)
C1—C6—N1	120.0 (3)	O2—C17—C16	107.9 (2)
C5—C6—N1	120.8 (3)	C18—C17—C16	112.9 (2)
O1—C7—N1	120.4 (3)	O2—C17—H17	110.4
O1—C7—C8	121.6 (3)	C18—C17—H17	110.4
N1—C7—C8	118.0 (3)	C16—C17—H17	110.4
C9—C8—C7	117.0 (3)	C19—C18—C17	101.2 (2)
C9—C8—S1	125.1 (3)	C19—C18—H18A	111.5
C7—C8—S1	117.5 (3)	C17—C18—H18A	111.5
C8—C9—C10B	140.4 (11)	C19—C18—H18B	111.5
C8—C9—C10A	128.0 (7)	C17—C18—H18B	111.5
C8—C9—H9	116.0	H18A—C18—H18B	109.3
C10A—C9—H9	116.0	N2—C19—C20	121.0 (3)
C11A—C10A—C15A	113.4 (15)	N2—C19—C18	113.8 (3)
C11A—C10A—C9	131.0 (14)	C20—C19—C18	125.1 (3)
C15A—C10A—C9	112.0 (14)	C21—C20—C25	118.2 (3)
C10A—C11A—C12A	120.2 (12)	C21—C20—C19	120.4 (3)
C10A—C11A—H11A	119.9	C25—C20—C19	121.4 (3)
C12A—C11A—H11A	119.9	C20—C21—C22	120.7 (3)
C13A—C12A—C11A	122.4 (13)	C20—C21—H21	119.7
C13A—C12A—H12A	118.8	C22—C21—H21	119.7
C11A—C12A—H12A	118.8	C23—C22—C21	120.2 (4)
C14A—C13A—C12A	118.2 (14)	C23—C22—H22	119.9
C14A—C13A—H13A	120.9	C21—C22—H22	119.9
C12A—C13A—H13A	120.9	C24—C23—C22	120.1 (4)
C13A—C14A—C15A	118 (2)	C24—C23—H23	120.0
C13A—C14A—H14A	121.0	C22—C23—H23	120.0
C15A—C14A—H14A	121.0	C23—C24—C25	120.3 (4)
C14A—C15A—C10A	120.5 (17)	C23—C24—H24	119.9
C14A—C15A—H15A	119.8	C25—C24—H24	119.9
C10A—C15A—H15A	119.8	C24—C25—C20	120.5 (4)
C15B—C10B—C9	125 (3)	C24—C25—H25	119.7
C15B—C10B—C11B	113 (3)	C20—C25—H25	119.7
C9—C10B—C11B	117 (2)	C7—N1—C6	124.2 (3)
C12B—C11B—C10B	121.4 (16)	C7—N1—C16	115.4 (2)
C12B—C11B—H11B	119.3	C6—N1—C16	119.8 (2)
C10B—C11B—H11B	119.3	C19—N2—O2	109.2 (2)
C11B—C12B—C13B	115.7 (17)	N2—O2—C17	108.8 (2)
C11B—C12B—H12B	122.1	C8—S1—C1	99.02 (15)
C13B—C12B—H12B	122.1		

### Hydrogen-bond geometry (Å, º)

**Table:**

*D*—H···*A*	*D*—H	H···*A*	*D*···*A*	*D*—H···*A*
C21—H21···O1^i^	0.93	2.43	3.339 (4)	166
C18—H18*B*···N2^ii^	0.97	2.56	3.526 (3)	178

Symmetry codes: (i) −*x*+1, −*y*, −*z*+1; (ii) *x*, *y*−1, *z*.
