# Supplementary figures and images for: Crystal structure of (E)-2-benzyl­idene-4-[(3-phenyl-4,5-di­hydro­isoxazol-5-yl)meth­yl]-2H-benzo[b][1,4]thia­zin-3(4H)-one
Source: Acta Crystallogr E Crystallogr Commun. 2015 May 23;71(Pt 6):o423–4. doi: 10.1107/S2056989015009755 (PMC4459330; doi:10.1107/S2056989015009755)

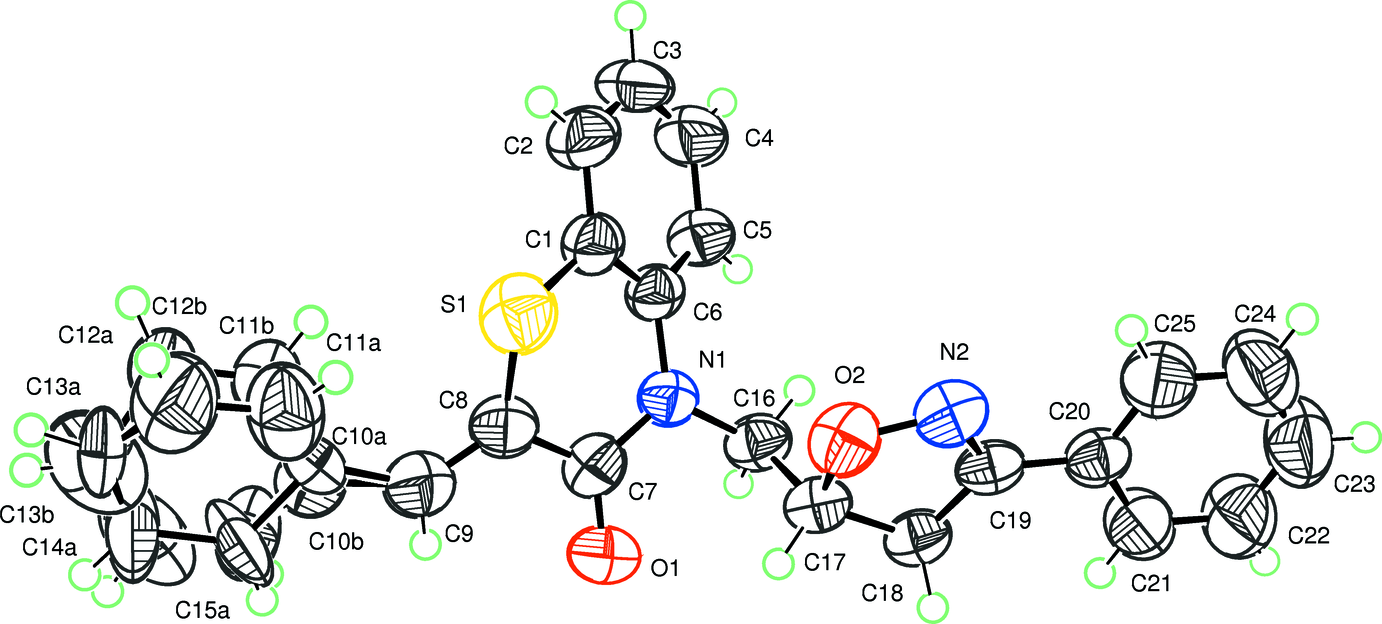

Supplement: Supplementary file 4 [file e-71-0o423-fig1.tif]
